# Case Report: Beyond commensal: *Staphylococcus epidermidis* as a novel cause of NARDS

**DOI:** 10.3389/fped.2026.1631683

**Published:** 2026-04-15

**Authors:** Linlin Chen, Jie Li, Xixi Liu, Xiyang Chen, Haiting Li, Dengpan Xie, Yunqin Chen, Junhui Yuan, Enfu Tao

**Affiliations:** Department of Neonatology and Neonatal Intensive Care Unit, Wenling Maternal and Child Health Care Hospital, Wenling, Zhejiang Province, China

**Keywords:** metagenomic next-generation sequencing, neonatal acute respiratory distress syndrome, neonatal sepsis, premature rupture of membranes, *Staphylococcus epidermidis*

## Abstract

*Staphylococcus epidermidis* (*S. epidermidis*), usually a harmless skin bacterium, can become an opportunistic pathogen in newborns, particularly those with risk factors like premature membrane rupture. Although it commonly causes late-onset sepsis, its association with neonatal acute respiratory distress syndrome (NARDS) is rare. This report describes a unique case of NARDS in a full-term newborn caused by *S. epidermidis*. The infant, born via cesarean at 40 2/7 weeks with a 30.5-hour membrane rupture, developed severe respiratory failure shortly after birth, necessitating mechanical ventilation. Initial treatment with penicillin and cefotaxime was ineffective, and by day 3, the infant's condition worsened, showing respiratory distress, petechial rashes, and high inflammatory markers. Treatment was changed to vancomycin and meropenem, with the addition of intravenous immunoglobulin and two doses of pulmonary surfactant. Metagenomic next-generation sequencing (mNGS) confirmed *S. epidermidis* in the airway secretions. The patient was discharged after 19 days with a diagnosis of NARDS, intrauterine infectious pneumonia, neonatal air leak syndrome, type II respiratory failure, neonatal sepsis, and congenital heart defects. In conclusion, *S. epidermidis* is a novel pathogen capable of causing NARDS in high-risk infants with prolonged membrane rupture. The proposed mechanisms—including surfactant dysfunction and biofilm-associated virulence—are supported by experimental literature and are consistent with the clinical phenotype observed in our patient, though direct confirmation requires further study. Notably, skin symptoms like erythematous rash and petechiae may indicate invasive *S. epidermidis* infection, especially in cases of respiratory distress with skin symptoms following premature rupture of membranes. Moreover, mNGS is vital for pathogen identification when traditional cultures fail.

## Introduction

Neonatal acute respiratory distress syndrome (NARDS) is a severe condition involving lung inflammation and low oxygen levels, leading to high illness and death rates in newborns (1). It involves surfactant dysfunction, lung inflammation, volume loss, increased shunting, and alveolar damage (1, 2).

NARDS can be triggered by direct lung insults like pneumonia and aspiration or indirectly by conditions such as sepsis, necrotizing enterocolitis, and chorioamnionitis (1). Sepsis is the leading cause of NARDS, responsible for 37.7% of cases, followed by aspiration events (27.2%) and pneumonia (27.2%) (3). Neonatal pneumonia, often caused by bacterial infections, can worsen respiratory distress and contribute significantly to NARDS (4). However, there is limited literature on the specific bacteria causing NARDS in bacterial pneumonia.

*Staphylococcus epidermidis (S. epidermidis),* typically a skin commensal, has been increasingly linked to pneumonia and sepsis in neonatal intensive care unit (NICU) (5, 6). However, cases of NARDS due to *S. epidermidis* are underreported. This paper discusses a rare case of NARDS from *S. epidermidis* infection, including its pathogenesis, diagnosis, and management.

## Case presentation

**Figure 1:**
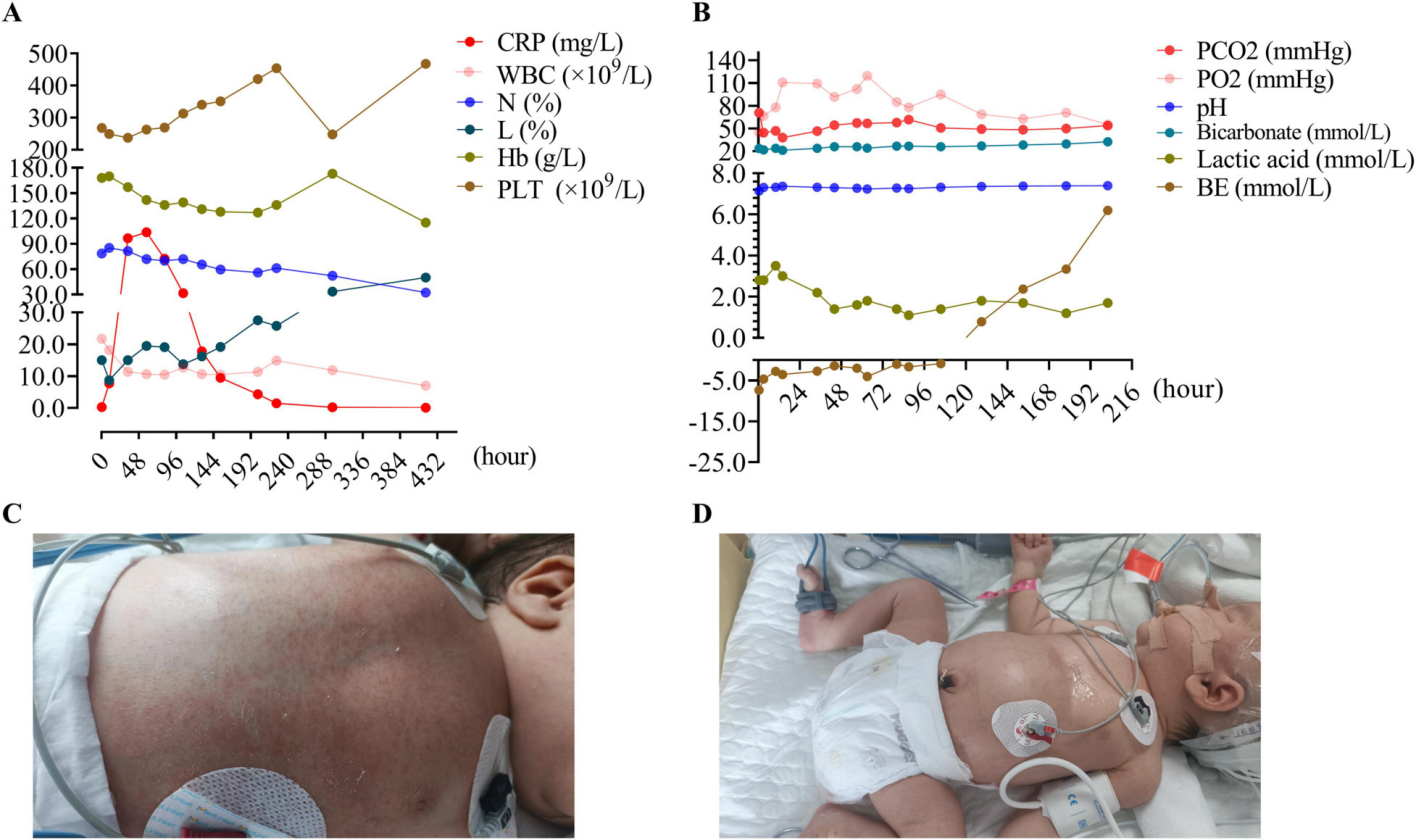
Laboratory findings and clinical course of the term neonate with *Staphylococcus epidermidis*-induced acute respiratory distress syndrome. **(A)** Complete blood count. Blood samples were collected at the following time points (hours post-admission): 0.4, 10, 34, 58, 81, 105, 129, 153, 201, 225, 297, 417, **(B)** arterial blood gas analysis. Blood samples were collected at the following time points (hours post-admission): 0.4, 3, 10, 14, 34, 44, 57, 63, 80, 87, 105.5, 129, 153, 178, 202. **(C)** Clinical photograph depicting the infant's diffuse erythematous rash and inguinal petechiae as observed on the third day of hospitalization. **(D)** Resolution of cutaneous manifestations.

**Figure 2:**
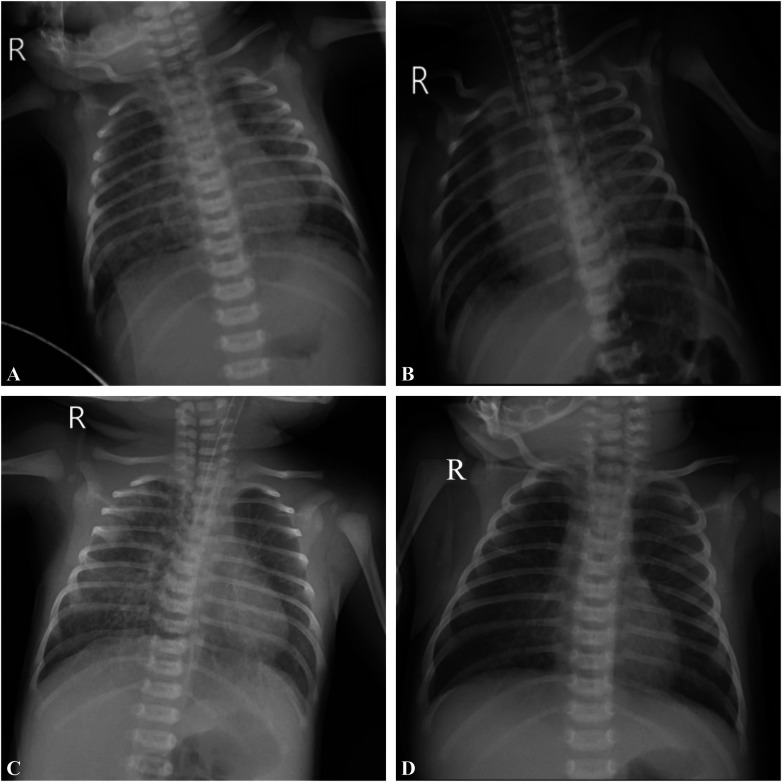
Representative imaging findings in term infants with neonatal acute respiratory distress syndrome during hospitalization. **(A)** Bedside chest x-ray after admission showing patchy opacities in the lungs. **(B)** Bedside chest x-ray on day 2 after admission revealing pneumothorax. **(C)** Bedside chest x-ray on day 3 after admission demonstrating multiple scattered patchy and linear opacities in both lung fields, along with progressive right lower lobe consolidation. **(D)** Bedside chest x-ray on day 18 after admission revealing almost complete resolution of lung infiltrates.

A female newborn delivered via cesarean at 40 2/7 weeks, weighing 3,480 g, had Apgar scores of 8 at 1 and 5 min. She developed respiratory depression and circumoral cyanosis 10 min after birth, necessitating NICU admission. Maternal risk factors included gestational diabetes, fever during labor, and a prolonged rupture of membranes, suggesting possible intrauterine infection. The infant showed tachypnea, nasal flaring, and intercostal retractions. Lab tests indicated leukocytosis with neutrophilia (Figure 1A), and arterial blood gas confirmed type II respiratory failure (Figure 1B). Chest x-ray showed patchy opacities in the lungs (Figure 2A), pointing to neonatal intrauterine infectious pneumonia. She was started on intravenous penicillin G (50,000 IU/kg q12 h) and cefotaxime (50 mg/kg q12 h).

The infant's respiratory condition deteriorated quickly, necessitating mechanical ventilation an hour after admission (initial settings: synchronized intermittent mandatory ventilation [SIMV] with peak inspiratory pressure [PIP] 20 cmH₂O, positive end-expiratory pressure [PEEP] 5 cmH₂O, fraction of inspired oxygen [FiO₂] 0.4). By 10 h of life, despite initial antibiotics, inflammatory markers continued to rise [high-sensitivity C-reactive protein (hs-CRP) 7.7 mg/L] and chest imaging showed progressive opacities, prompting replacement of cefotaxime with cefoperazone-sulbactam (40 mg/kg q12 h) to enhance coverage against Gram-negative organisms. By day 2, a pneumothorax was detected (Figure 2B), prompting transition to high-frequency oscillatory ventilation (HFOV) starting at 24 h of life. HFOV was continued for approximately three days (from 24 to 104 h of life), with mean airway pressure titrated between 13 and 16 cmH₂O, amplitude 30–45, and FiO₂ ranging from 0.5 to 0.6. Concurrently, inlfammatory markers rose (Figure 1A), prompting an antibiotic change from penicillin and cefoperazone-sulbactam to meropenem. On day 3, the clinical picture evolved further with the appearance of a scattered erythematous rash and inguinal petechiae (Figure 1C), accompanied by a sharp increase in C-reactive protein (CRP), strongly suggestingneonatal sepsis (Figure 1A). This prompted the addition of vancomycin and intravenous immunoglobulin (IVIG). Despite negative blood culturesa comprehensive diagnostic work-up was conducted to identify the underlying etiology. On day 3, metagenomic next-generation sequencing (mNGS) of tracheal secretions was performed using an Illumina platform with a detection limit of 50–500 copies/mL (total reads 29 million, Q30 95.8%) (7). Bioinformatic analysis against a database of 18,562 microbial genomes detected *S. epidermidis* at 8.3% relative abundance (11 total reads) and *Corynebacterium propinquum* at 5.3% relative abundance (7 reads), with no other bacterial, fungal, or viral pathogens identified. Concurrent chest imaging (Figure 2C) revealed worsening pulmonary involvement-multiple scattered patchy and linear opacities in both lung fields, along with progressive right lower lobe consolidation- and with worsening ventilator requirements (oxygenation index = 8.2), indicating moderate NARDS (2). In response, a 2-day surfactant therapy resulted in rapid clinical improvement, with complete rash resolution within 24 h (Figure 1D) and decreasing inflammatory markers (Figure 1A). Cardiovascular assessment revealed an atrial septal defect (ASD), patent ductus arteriosus (PDA), and mild pulmonary hypertension, which were managed with sildenafil and dopamine. Additional investigations, including electrolyte panels, glucose monitoring, hepatic/renal function tests, thyroid studies, TORCH serology, cranial/abdominal ultrasonography, and amplitude-integrated electroencephalography (aEEG), all returned normal results. The patient progressively recovered, successfully extubated after one week, transitioned to nasal continuous positive airway pressure (NCPAP) by day 12, and discontinued supplemental oxygen by day 18. A chest x-ray showed nearly complete resolution of lung infiltrates (Figure 2D), with normalized inflammatory markers (Figures 1A,B) and stable cardiorespiratory status. The infant was discharged on day 19.

A follow-up echocardiogram at 30 months showed complete resolution of cardiac anomalies, indicating normal cardiac anatomy and function. Additionally, the child showed normal growth and achieved all expected neurodevelopmental milestones.

## Discussion

This case of *S. epidermidis*-induced NARDS highlights the pathogenic potential of this usually harmless skin bacterium in NICU environments. While *S. epidermidis* typically colonizes the skin and mucosal surfaces (8–11), it can lead to pneumonia, sepsis (12, 13), and now NARDS, as evidenced by our patient's case, the first reported instance of NARDS caused by this bacterium. Several lines of evidence support *S. epidermidis* as the causative pathogen in this infant. First, the temporal association was compelling: clinical deterioration with respiratory failure and systemic inflammation occurred in parallel with the detection of *S. epidermidis* by mNGS on day 3. Second, the microbiological profile was strikingly paucimicrobial—airway secretions yielded *S. epidermidis* (8.3% relative abundance, 11 reads) and a single low-pathogenicity commensal (*Corynebacterium propinquum*), with no other bacterial, fungal, or viral pathogens identified. Third, the appearance of a characteristic erythematous rash and inguinal petechiae on day 3, coinciding with peak inflammatory markers, provided a clinical signature previously associated with invasive *S. epidermidis* infection in neonates (14, 15). Fourth, the rapid and sustained clinical response to vancomycin—rash resolution within 24 h and normalization of inflammatory markers—offers *post-hoc* evidence of susceptibility and causation. Collectively, these observations establish *S. epidermidis* as a genuine pathogen in this case of NARDS. This case is notable precisely because it deviates from the typical presentation of neonatal coagulase-negative staphylococci (CoNS) infections, demonstrating that *S. epidermidis* can, in certain high-risk contexts, cause severe and atypical disease phenotypes such as NARDS.

While our case does not provide direct molecular evidence for specific pathogenic pathways, the clinical phenotype described above is consistent with several well-documented virulence mechanisms of *S. epidermidis* in the experimental literature. Drawing on these studies, we briefly summarize how this commensal organism might induce lung injury. Surfactant inactivation is critical in NARDS related to pulmonary infections (4), and Griese et al. found that *S. epidermidis* alters neonatal RDS surfactant in several ways, including reducing phospholipid mass in airway samples and changing the surfactant aggregate ratios, indicating impaired surfactant metabolism (16). *In vitro* studies showed that *S. epidermidis* triggers pro-inflammatory responses in lung cells, increasing mediators like tumor necrosis factor (TNF)-*α*, interleukin (IL)-1β, IL-6, IL-8, monocyte chemoattractant protein (MCP)-1, interferon gamma-induced protein 10 (IP-10), and intercellular adhesion molecule (ICAM)-1 (17). Immune activation fragments surfactant components and proteins, exacerbated by Toll-like receptor and complement pathway activation from inflammatory compounds. This leads to surfactant degradation and impaired lung function due to reduced surface tension-lowering ability (18). Additionally, *S. epidermidis* activates immune responses via its peptidoglycan cell wall components, engaging pattern recognition receptors (RPRs) and increasing pro-inflammatory cytokines and chemokines, which exacerbates inflammation (19). Moreover, *S. epidermidis* interacts with host tissues via surface proteins like the accumulation-associated protein (Aap), which promotes adherence to skin corneocytes. This glycan-dependent adherence aids colonization and may trigger the host's immune response, potentially causing inflammation (8). The bacterium also forms biofilms, a major virulence factor linked to its peptidoglycan cell wall, which activates the immune system. Biofilms stimulate phagocytic cells, such as polymorphonuclear neutrophils (PMNs), to attach, phagocytose, and release bactericidal substances, although excessive inflammation can result if unchecked (20). Additionally, *S. epidermidis* alters its peptidoglycan and biofilm matrix when interacting with human platelets, increasing resistance to immune responses and antimicrobial agents (21). These experimental observations provide a plausible biological framework for understanding the severity of our patient's illness. The convergence of surfactant dysfunction, intense pulmonary inflammation, and biofilm-forming capacity could explain the rapid progression to moderate NARDS (oxygenation index 8.2) despite initial broad-spectrum antibiotics, as well as the dramatic clinical improvement following targeted vancomycin therapy. We emphasize, however, that direct confirmation of these mechanisms in human neonates awaits further investigation.

Diagnosing *S. epidermidis* infections in neonates is challenging due to its dual role as a commensal and pathogen. As a common skin colonizer, it complicates the differentiation between contamination and true infection, particularly in the NICU, where it is a major cause of late-onset sepsis in preterm infants (12). Its prevalence is linked to indwelling medical devices that facilitate biofilm formation, leading to increased antibiotic resistance and immune evasion (22). Moreover, *S. epidermidis* exhibits significant genetic diversity in the NICU, resulting in varying virulence and resistance patterns (9). Despite its clinical importance, blood cultures have low sensitivity for detecting *S. epidermidis*, with an average positivity rate of only 2.50% over five years in China, where it is the most common isolate among coagulase-negative *staphylococci* (13, 23)*.* Blood cultures were negative in our case, but clinical signs suggested an infection. mNGS enabled unbiased detection of microbial nucleic acids (24, 25), confirming *S. epidermidis* infection in airway secretions. This led to timely antibiotic adjustments and clinical improvement. While mNGS cannot replace antimicrobial susceptibility testing (AST), it enhances pathogen detection and provides important diagnostic insights (26). In this case, because the pathogen was identified solely by mNGS without a corresponding cultured isolate, formal AST could not be performed. We acknowledge this as an inherent limitation of culture-independent diagnostics. However, the rapid and sustained clinical improvement following vancomycin therapy—including complete rash resolution within 24 h and normalization of inflammatory markers—provides indirect evidence of vancomycin susceptibility. This clinical inference, while supportive, does not substitute for formal AST, and future cases would benefit from concurrent culture-based susceptibility testing whenever possible. This case underscores mNGS's value in diagnosing infectious NARDS when traditional methods fall short. An additional diagnostic consideration in this case is the need to exclude cardiac failure as the primary cause of respiratory distress, as required by the Montreux definition of NARDS (2). The presence of an ASD and PDA raises the question of whether left-to-right shunting could account for the observed oxygenation disturbance. Several observations support NARDS as the primary diagnosis rather than cardiac failure. First, the temporal profile—respiratory failure peaked in concert with infectious signs (rash, elevated CRP on day 3) and improved with targeted antibiotics—aligns more closely with infection-induced lung injury than with fixed cardiac shunting. Second, the rapid clinical response to antibiotic change from vancomycin and surfactant, including rash resolution within 24 h, would be atypical for pure cardiac failure. Third, the infant remained hemodynamically stable with medical management (sildenafil and dopamine), suggesting that the left-to-right shunt was not the dominant driver of oxygenation failure. Thus, while these cardiac anomalies are noted, they do not fulfill the Montreux exclusion criterion of ‘respiratory failure fully explained by cardiac failure’ (2).

While mNGS offered clear advantages in pathogen detection in this case, several limitations of this technology must be acknowledged (24, 25, 27). First, the high sensitivity of mNGS makes it susceptible to contamination from skin flora or environmental sources, particularly in non-sterile specimens such as tracheal aspirates (24, 25). In our case, the detection of *Corynebacterium propinquum*—a common respiratory commensal—illustrates this challenge, and careful interpretation is required to distinguish true pathogens from background microbiota. Second, mNGS cannot reliably differentiate between colonization and active infection; this distinction relies on clinical correlation, as we have emphasized throughout this case (26, 27). Third, the technical complexity of mNGS, including DNA extraction, library preparation, and bioinformatic analysis, requires specialized expertise and infrastructure, limiting its availability in many settings (27). Fourth, the cost of mNGS remains substantially higher than conventional cultures, which may restrict its use as a first-line diagnostic tool (24, 25). Finally, the turnaround time—typically 24–48 h in clinical practice—is longer than that of Gram stain or rapid antigen tests, though it is comparable to culture (24, 27). Despite these limitations, mNGS proved invaluable in this case, where conventional methods failed to identify the causative pathogen.

For infection-induced NARDS, treatment targets the underlying cause. In cases of neonatal infection following premature membrane rupture, broad-spectrum antibiotics are essential. In China, the leading pathogens for early-onset sepsis (EOS) are *Escherichia coli* (*E. Coli*), *Group B Streptococcus* (GBS), and *Klebsiella pneumoniae*, with *Klebsiella* more common in preterm infants (28). We initially used penicillin and cefotaxime for GBS and *E. coli,* but after no improvement in inflammatory markers, we switched to penicillin and cefoperazone. As infection markers worsened, we escalated treatment to penicillin and meropenem. By the third postnatal day, the infant's condition deteriorated, prompting the addition of vancomycin and IVIG.

This case highlights the challenges of selecting antibiotics for neonates. While narrow-spectrum antibiotics are ideal for infection prevention, low blood culture positivity rates complicate pathogen identification. The emergence of rashes on day 3 raised suspicions of gram-positive cocci, later confirmed by mNGS. This suggests that, despite common pathogens in EOS, other organisms like *S. epidermidis* should be considered, especially with erythematous rashes or petechiae. The case offers insights into managing neonatal infections, noting that while surfactant therapy is not routinely recommended for NARDS, it can be beneficial in select cases (3, 29). However, NARDS often poorly responds to surfactant due to inflammatory mediators (1). The infant received two doses of pulmonary surfactant, targeted antibiotics, and IVIG, resulting in infection control, rash resolution, and respiratory improvement, with recovery largely attributed to timely antibiotic administration. A Chinese multicenter study indicated that multiple surfactant doses increased mortality compared to single or no doses, raising concerns about its use in NARDS and suggesting caution with repeat doses (30). Recent research suggests that surfactant-budesonide combination therapy may be effective for NARDS resulting from late-onset neonatal sepsis (14, 31). Further clinical studies are necessary to investigate the therapeutic potential of surfactant alone or with budesonide for *S. epidermidis*-induced NARDS.

Interestingly, on the third day of hospitalization, the infant exhibited scattered erythematous rashes on the trunk and petechiae in the bilateral inguinal regions, alongside a notable increase in high-sensitivity C-reactive protein (hs-CRP), suggesting a possible infection. This skin presentation may result from *S. epidermidis* invading the skin and soft tissues, causing localized inflammation and microvascular damage. Notably, *S. epidermidis* has been associated with severe necrotizing soft tissue infections, including necrotizing fasciitis—a life-threatening condition characterized by rapid necrosis of fascial and subcutaneous tissues (15). Additionally, coagulase-negative staphylococci (CoNS), particularly *S. epidermidis*, are commonly found on the skin of healthy 1-day-old neonates, even in cases of erythema toxicum neonatorum (32). This observation indicates that while *S. epidermidis* usually colonizes neonatal skin harmlessly, it can become pathogenic in immunocompromised individuals, leading to tissue invasion and inflammation. The infection likely arises from maternal-fetal transmission after premature rupture of membranes, a known risk factor for *S. epidermidis* infection (10). Bacterial translocation may cause complications like neonatal sepsis (33), NEC (34), or severe systemic infections (35). In this case, the pathogen's spread to the lungs may have led to NARDS (33). The rash observed could result from either direct bacterial proliferation or indirect inflammatory damage from systemic cytokine release.

While specific virulence gene analysis was not performed from the mNGS data in this clinical case——as this was beyond the scope of routine diagnostic testing——the existing literature implicates several well-established virulence mechanisms of *S. epidermidis* that may have contributed to NARDS. Future studies incorporating targeted virulence profiling from mNGS data could provide further insights into pathogen-host interactions in neonatal infections and may ultimately guide more personalized therapeutic strategies.

## Conclusion

This case underscores *S. epidermidis* as a potential pathogen causing NARDS in high-risk infants with prolonged membrane rupture. The challenges of diagnosing culture-negative sepsis highlight the value of mNGS for timely treatment. Skin manifestations may serve as early signs of infection. Further research is needed to enhance surfactant and adjunctive therapies for *S. epidermidis*-induced NARDS.

## Data Availability

The original contributions presented in the study are included in the article/Supplementary Material, further inquiries can be directed to the corresponding authors.
